# Highly Sensitive MoS_2_–Indocyanine Green Hybrid for Photoacoustic Imaging of Orthotopic Brain Glioma at Deep Site

**DOI:** 10.1007/s40820-018-0202-8

**Published:** 2018-04-23

**Authors:** Chengbo Liu, Jingqin Chen, Ying Zhu, Xiaojing Gong, Rongqin Zheng, Ningbo Chen, Dong Chen, Huixiang Yan, Peng Zhang, Hairong Zheng, Zonghai Sheng, Liang Song

**Affiliations:** 1 Research Laboratory for Biomedical Optics and Molecular Imaging, Shenzhen Key Laboratory for Molecular Imaging, Institute of Biomedical and Health Engineering, Shenzhen Institutes of Advanced Technology, Chinese Academy of Sciences, Shenzhen, 518055 People’s Republic of China; 2 Shenzhen College of Advanced Technology, University of Chinese Academy of Sciences, Shenzhen, 518055 People’s Republic of China; 3 Department of Medical Ultrasound, The Third Affiliated Hospital of Sun Yat-Sen University, Guangzhou, 510630 People’s Republic of China; 4 Paul C. Lauterbur Research Center for Biomedical Imaging, Institute of Biomedical and Health Engineering, Shenzhen Institutes of Advanced Technology, Chinese Academy of Sciences, Shenzhen, 518055 People’s Republic of China; 5 Translational Medicine R&D Center, Institute of Biomedical and Health Engineering, Shenzhen Institutes of Advanced Technology, Chinese Academy of Sciences, Shenzhen, 518055 People’s Republic of China

**Keywords:** MoS_2_–ICG hybrid, Orthotopic brain glioma, Photoacoustic imaging, Molecular imaging

## Abstract

Photoacoustic technology in combination with molecular imaging is a highly effective method for accurately diagnosing brain glioma. For glioma detection at a deeper site, contrast agents with higher photoacoustic imaging sensitivity are needed. Herein, we report a MoS_2_–ICG hybrid with indocyanine green (ICG) conjugated to the surface of MoS_2_ nanosheets. The hybrid significantly enhanced photoacoustic imaging sensitivity compared to MoS_2_ nanosheets. This conjugation results in remarkably high optical absorbance across a broad near-infrared spectrum, redshifting of the ICG absorption peak and photothermal/photoacoustic conversion efficiency enhancement of ICG. A tumor mass of 3.5 mm beneath the mouse scalp was clearly visualized by using MoS_2_–ICG as a contrast agent for the in vivo photoacoustic imaging of orthotopic glioma, which is nearly twofold deeper than the tumors imaged in our previous report using MoS_2_ nanosheet. Thus, combined with its good stability and high biocompatibility, the MoS_2_–ICG hybrid developed in this study has a great potential for high-efficiency tumor molecular imaging in translational medicine.

**Electronic supplementary material:**

The online version of this article (10.1007/s40820-018-0202-8) contains supplementary material, which is available to authorized users.

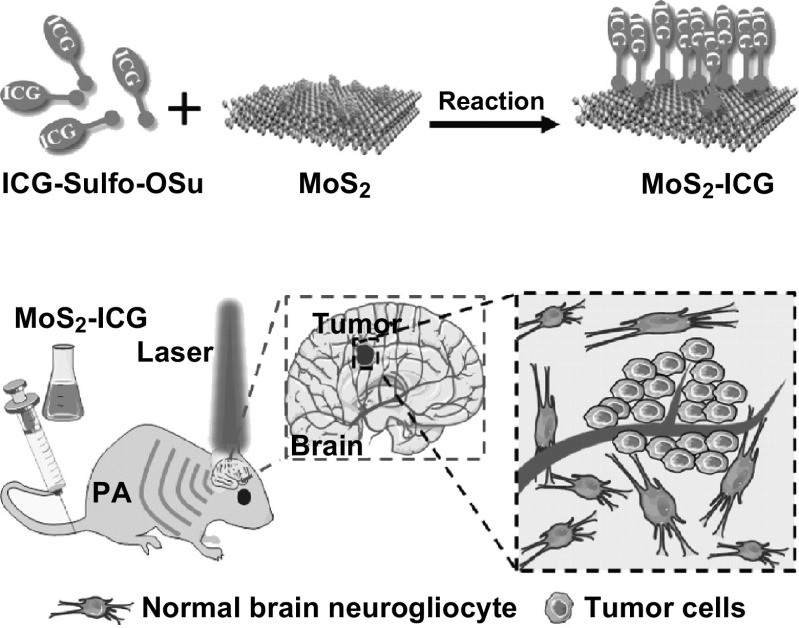

## Highlights

- MoS_2_ nanosheets was covalently conjugated with indocyanine green (ICG) by facile mixing ICG-Sulfo-NHS and MoS_2_ nanosheets.
- The 3.5 mm imaging depth demonstrated in this study is one of the deepest among all the glioma photoacoustic imaging research reported so far by using the nanoprobe in the NIR I spectral region.
- The design and validation of the MoS_2_–ICG hybrid bring up an effective strategy for synthesizing highly sensitive photoacoustic nanoprobes, i.e., by covalently conjugating optical dyes with transition metal dichalcogenides.

## Introduction

Brain glioma is a highly invasive intracranial tumor that accounts for nearly one-third of all tumor cases in the central nervous system and shows a high incidence rate, high mortality rate, high recurrence rate, and low curing rate [1, 2]. The 5-year survival rate of glioma for adults is less than 5% [3]. Invasive growth of glioma cells obscures the boundary between normal brain tissue and tumor tissue, making it extremely difficult to accurately diagnose this tumor and delineate the tumor boundary [4]. Novel imaging methods with high sensitivity, specificity, and resolution at tumor-relative imaging depth are urgently needed for accurate diagnosis of glioma, based on which precise tumor surgery can be performed to achieve complete tumor removal and improve the prognosis of the patients [5, 6].

As a novel non-ionizing imaging method, photoacoustic imaging has been rapidly developed in recent years [7, 8]. This imaging technology acquires structural, functional, and molecular information for biological tissues by detecting the acoustic signals generated from the chromophores in the tissue [9–11]. As the contrast of photoacoustic imaging originates from the discrepancy of optical absorption, it retains the high sensitivity and specificity of conventional optical imaging methods [12–14]. Moreover, by detecting the significantly less scattered acoustic waves, photoacoustic imaging can achieve better resolution at higher imaging depth in biological tissues [15–17]. However, when used for brain glioma detection, the lack of endogenous contrast is a major limitation to the distinction between tumor and normal tissues through photoacoustic imaging.

The advent of molecular imaging has provided unprecedented opportunities for glioma detection with high sensitivity and specificity, as the tumor cells can be selectively labeled with exogenous contrast agents to achieve tumor-specific enhanced imaging. Hence, by combining photoacoustic imaging with molecular imaging (i.e., photoacoustic molecular imaging), brain glioma can be diagnosed with high sensitivity, specificity, and resolution at greater depth [18–20]. Nanomaterials such as gold nanoparticles and organic nanoparticles have been used as contrast agents for photoacoustic imaging of glioma [20, 21]. While these contrast agents have good photoacoustic imaging effects, the relatively low near-infrared (NIR) absorbance, narrow NIR absorption spectrum, and short absorbance–wavelength limit their potential for highly efficient in vivo photoacoustic imaging applications. Kircher et al. [20] first synthesized a photoacoustic-magnetic resonance imaging (MRI)-Raman triple-modality imaging nanoprobe and applied it to delineate brain glioma margins. The optical absorption peak of the nanoprobe is approximately 540 nm, which is very close to that of endogenous hemoglobin. Therefore, it is difficult to distinguish the photoacoustic signal of the nanoprobe from that of hemoglobin in in vivo imaging applications. Fan et al. [21] fabricated a perylene bisimide-based nanoparticle with a 675-nm absorbance peak for in situ photoacoustic imaging of mouse C6 brain glioma. The nanoparticle was shown to have excellent enhanced permeability and retention effects for tumor passive targeting. However, as an organic functional small molecule, perylene bisimide tends to be cleared from the blood circulation quickly. Moreover, the absorbance peak of perylene bisimide at 675 nm is close to visible light range, which may lead to high background photoacoustic signals in the blood. Therefore, it is of great needs to develop exogenous probe with long absorption wavelength and high sensitivity for photoacoustic imaging of orthotopic brain glioma.

Molybdenum disulfide (MoS_2_), a kind of transition metal dichalcogenides with distinctive physical and chemical properties, has sparked an explosion of interest in biomedicine [22–24]. In our previous study, a MoS_2_ nanoplatform was synthesized and shown to have with good biocompatibility and excellent tumor targeting capability for orthotopic glioma imaging [22]. In situ brain glioma sitting 1.8 mm beneath the mouse scalp was clearly visualized with the aid of MoS_2_ nanosheet-enhanced photoacoustic imaging. However, the imaging depth must be further increased for glioma detection at even deeper sites. Hence, contrast agents with higher photoacoustic imaging sensitivity (in specific, longer absorption wavelength and higher absorption coefficient) are needed. Because of its large specific surface area, MoS_2_ nanosheets have a high loading capability, providing an effective strategy for synthesizing highly sensitive photoacoustic contrast agents by loading the optical dyes onto its surface [23–27]. Once loaded onto the MoS_2_ nanosheets surface, the optical dye is stabilized, and its internal blood circulation time is significantly prolonged. This high loading capability of MoS_2_ nanosheets and synergetic absorbance of optical dye along with MoS_2_ nanosheets endow the agent with a high optical absorption characteristic, making it a suitable candidate for photoacoustic molecular imaging applications.

**Scheme 1:**
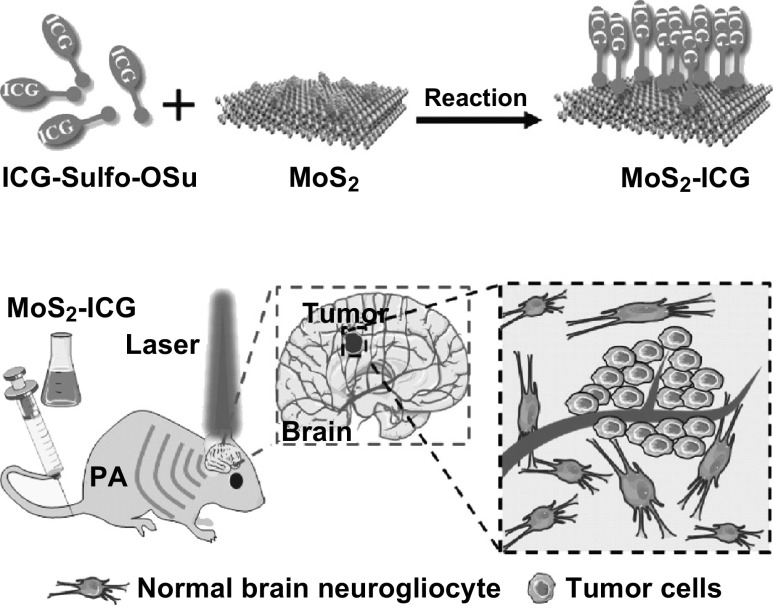
Schematic of MoS_2_–ICG hybrid synthesis and its application in photoacoustic (PA) imaging of orthotopic brain glioma

Herein, we developed a covalently conjugating strategy for the synthesis of a MoS_2_–indocyanine green (ICG) hybrid for in vivo photoacoustic imaging of orthotropic glioma at deep site (Scheme 1). ICG is an FDA-approved optical dye with a high NIR extinction coefficient [28, 29]. By conjugating ICG with MoS_2_ nanosheets, the absorption peak of MoS_2_–ICG is redshifted to 800 nm, much longer than that of MoS_2_ nanosheets (800 *vs* 675 nm), enabling photoacoustic imaging at longer wavelength to achieve greater penetration depth and lower background noise [27, 30, 31]. In vitro studies were carried out to validate the optical and photoacoustic performance of MoS_2_–ICG by comparing it with MoS_2_ in our previous study [22]. Next, the hybrid was applied for in vivo photoacoustic imaging of orthotopic glioma, and the high sensitivity in deep glioma visualization was demonstrated.

## Experimental

### Materials

MoS_2_ was purchased from Sigma-Aldrich (St. Louis, MO, USA). Bovine serum albumin (BSA) was obtained from Biosharp (Seoul, Korea). Dulbecco’s modified Eagle’s medium (DMEM), fetal bovine serum (FBS), trypsin–EDTA solution, and phosphate-buffered saline (PBS) were purchased from Gibco (Grand Island, NY, USA). Cell counting kit-8 (CCK-8) and 4′,6-diamidino-2-phenylindole (DAPI) were obtained from Dojindo (Tokyo, Japan). ICG-Sulfo-OSu was obtained from AAT Bioquest, Inc. (Sunnyvale, CA, USA).

### BSA-Assisted Exfoliation of Monolayer MoS_2_ Nanosheets

Monolayer MoS_2_ nanosheets were exfoliated by ice-bath sonication in a solution of BSA and water. Briefly, 50 mg of MoS_2_ powder was added to 10 mL of an aqueous solution containing 10 mg of BSA. The mixed suspension was sonicated in an ice bath for 8 h. After centrifugation at 8000 rpm for 20 min, the supernatant was collected to obtain the monolayer MoS_2_ nanosheets.

### Synthesis of ICG-Conjugated MoS_2_ Nanosheets (MoS_2_–ICG)

Monolayer MoS_2_ nanosheets were covalently conjugated to ICG-Sulfo-NHS, an ICG derivative. Briefly, 1 mg ICG-Sulfo-NHS powder was dissolved in DMSO, and then added to 1 mL MoS_2_ nanosheets solution (~ 1 mg mL^−1^) and stirred at 25 °C for 12 h. Unbound ICG-Sulfo-NHS was removed by dialysis (8000–12,000 molecular cutoff) in deionized water for 24 h. The MoS_2_–ICG hybrid was collected and stored at 4 °C. All procedures were carried out without any direct light exposure. To determine the ICG loading efficiency, the optical absorbance of the dialysate was measured with a UV–Vis spectrometer at 780 nm and compared against a calibration curve to calculate the amount of unbound ICG. Conjugated ICG in MoS_2_ nanosheets was determined as unbound ICG subtracted from the total ICG used. The loading efficiency was calculated as *W*_1_/*W*_2_  ×  100%, where *W*_1_ represents the weight of the conjugated ICG in MoS_2_ nanosheets and *W*_2_ is the weight of MoS_2_.

### Characterization

Atomic force microscopy (AFM) images were captured with a Bruker microscope (Billerica, MA, USA). Fourier transform infrared (FTIR) spectra measurement was carried out using an FTIR spectrometer (Bruker Vertex 80 V). UV–Vis–NIR spectra were detected with a UV–Vis–NIR spectrophotometer (PerkinElmer Lambda 750, Waltham, MA, USA). Fluorescence spectra were measured with a Luminescence Spectrometer (LS 55, Perkin–Elmer). Cell viability was detected by a multimode reader (BioTek SynergyTM 4, Winooski, VT, USA). The concentration of MoS_2_ was measured by ICP-OES (PE ICP-OES Optima 7000DV, PerkinElmer, USA). Our custom-built acoustic resolution photoacoustic microscopy (AR-PAM) system was used for all photoacoustic measurements. The AR-PAM system consists of a tunable pulsed optical parametric oscillator (OPO) laser (Vibrant 355 II HE, Opotek, Carlsbad, USA), a focused ultrasound transducer (V315-SU, Olympus IMS, Waltham, USA; central frequency: 10 MHz; fractional bandwidth: 6 MHz; N.A.: 0.4), and a precision motorized 3D scanning stage (PSA2000-11, Zolix, Beijing, China).The lateral resolution of AR-PAM was measured to be 220 μm (theoretically calculated to be 210 μm) and the imaging depth reached ~ 10 mm as demonstrated in our previous study [29]. Detailed information regarding the system is given in supplementary information (Fig. S1).

### Cell Line and Animal Model

The human brain glioma cell line U87 was obtained from American Type Culture Collection (Manassas, VA, USA) and cultured in DMEM media supplemented with 10% FBS and 1% penicillin–streptomycin solution in a humidified incubator (5% CO_2_ at 37 °C). Balb/c nude mice (3–5 weeks old) were purchased from the Medical Experimental Animal Center of Guangdong Province (Guangzhou, China). For orthotopic glioma model establishment, 1 × 10^6^ U87 tumor cells in 6 μL PBS were injected into the striatum: Bregma 2.0 mm, left lateral 2.0 mm, depth 3.4 mm. The tumor growth was monitored by MRI (3T Magnetom Trio, Erlangen, Germany). All animal handling and experimental procedures were approved by the Animal Study Committee of Shenzhen Institutes of Advanced Technology, Chinese Academy of Sciences.

### Cellular Uptake of MoS_2_–ICG

Fluorescence imaging was applied to confirm the internalization of MoS_2_–ICG into tumor cells. U87 cells were first cultured in a confocal dish for 24 h, and then mixed with MoS_2_–ICG or free ICG (~ 0.2 mg mL^−1^) for 1, 3, and 8 h incubation. Free MoS_2_ nanosheets were removed from the cells by washing three times with PBS. The treated cells were fixed with paraformaldehyde solution (4%) for 8 min followed by DAPI (10 μg mL^−1^) for 3 min. Fluorescence images of the cells were captured with a Leica TCS SP5 confocal laser scanning microscope (Wetzlar, Germany; *E*_*x*_ = 405 and 633 nm for DAPI and ICG, respectively).

### In Vitro Biocompatibility

For the cytotoxicity assay, U87 cells were plated in a 96-well plate (5 × 10^4^ cells per well) and cultured for 24 h. Next, the cells were treated with different concentrations of MoS_2_–ICG (0.1, 0.2, 0.5, and 1 mg mL^−1^) for 24 h. Relative cell viability (RCV) was assessed by CCK-8 assay and then determined in a 96-well plate reader (BioTek Synergy™ 4) at 450 nm with Eq. 1:

1 $${\text{RCV }}\left( \% \right) = \frac{{A_{\text{t}} - A_{\text{nc}} }}{{A_{\text{pc}} - A_{\text{nc}} }} \times 100{\text{\% }}$$

where *A*_t_, *A*_pc_, and *A*_nc_ represent the absorbance of the tested group, positive control, and negative control, respectively. In addition, the whole blood of Balb/c nude mice was collected and centrifuged (1500 rpm, 3 min) to separate the red blood cells (RBCs). The RBCs were further washed with PBS three times. Next, 10% RBCs (v/v, in PBS) were incubated with different concentrations of MoS_2_–ICG (25, 50, 100, 150, 200, and 300 μg mL^−1^) at 37 °C for 3 h. After centrifugation (10,000 rpm, 1 min), the supernatant was collected and analyzed with a UV–Vis–NIR spectrometer at 541 nm. The hemolytic percentage (HP) is calculated using Eq. 2:

2 $${\text{HP }}\left( \% \right) = \frac{{D_{\text{t}} - D_{\text{nc}} }}{{D_{\text{pc}} - D_{\text{nc}} }} \times 100{\text{\% }}$$

where *D*_t_, *D*_pc_, and *D*_nc_ are the absorbance of the tested sample and the positive (deionized water) and negative (PBS) controls, respectively.

### In Vitro Photoacoustic Measurement

Monolayer MoS_2_ and MoS_2_–ICG water solutions containing the same concentration of MoS_2_ or different concentrations (1, 0.5, 0.25, and 0.125 mg mL^−1^) of MoS_2_–ICG were mixed with agarose gel (1.5%) at a 1:1 ratio and measured by AR-PAM under 800 nm laser irradiation to characterize their photoacoustic performance. The MoS_2_–ICG sample with the highest concentration was further covered with a mouse skull, and the photoacoustic cross-sectional B-scan images of the sample were obtained under 675- and 800-nm pulsed laser excitation, respectively. The pulse energy at both wavelengths before and after penetrating the skull was recorded with an energy meter (Nova II, Ophir, Jerusalem, Israel). Furthermore, MoS_2_–ICG was mixed (at 0–4 °C) with Matrigel matrix at a 1:1 ratio and then injected into the lower flank of mouse subcutaneously. After the sample mixture solidified, the injected region was imaged by AR-PAM under 675- and 800-nm pulsed laser excitation. The signal-to-noise ratio (SNR) was calculated as Eq. 3:

3 $${\text{SNR }} = \, 20 \, \times \, l \, g \, \left( {S/N} \right)$$

where *S* is the signal and *N* is the noise.

### In Vivo Photoacoustic Imaging of Orthotopic Glioma

Mice bearing orthotopic U87 glioma were anesthetized with a 2% isoflurane/oxygen mixture and placed in the prone position. Photoacoustic imaging of the tumor region was performed by AR-PAM under 800-nm laser irradiation before and at 1, 3, and 5 h post-intravenous injection of MoS_2_–ICG (100 μL, 1 mg mL^−1^). Ultrasound images of the tumor region were captured simultaneously with AR-PAM.

### In Vivo Biocompatibility of MoS_2_–ICG

Healthy Balb/c mice (5 mice in total) were intravenously injected with 10 mg kg^−1^ MoS_2_–ICG (150 μL). At days 1 and 15 post-injection, mouse blood was collected via orbit for complete blood panel testing at Shenzhen Center for Disease and Prevention. Furthermore, major organs from the MoS_2_–ICG-treated mice were harvested, including the heart, liver, kidney, lung, and spleen. The organ tissues were stained with hematoxylin and eosin (H&E) and examined under a digital microscope (Olympus, CX31, Tokyo, Japan) after fixation in 10% neutral-buffered formalin, embedding into paraffin and sectioning at 5 mm thickness.

## Results and Discussion

### Synthesis and Characterization of MoS_2_–ICG Hybrid

**Fig. 1:**
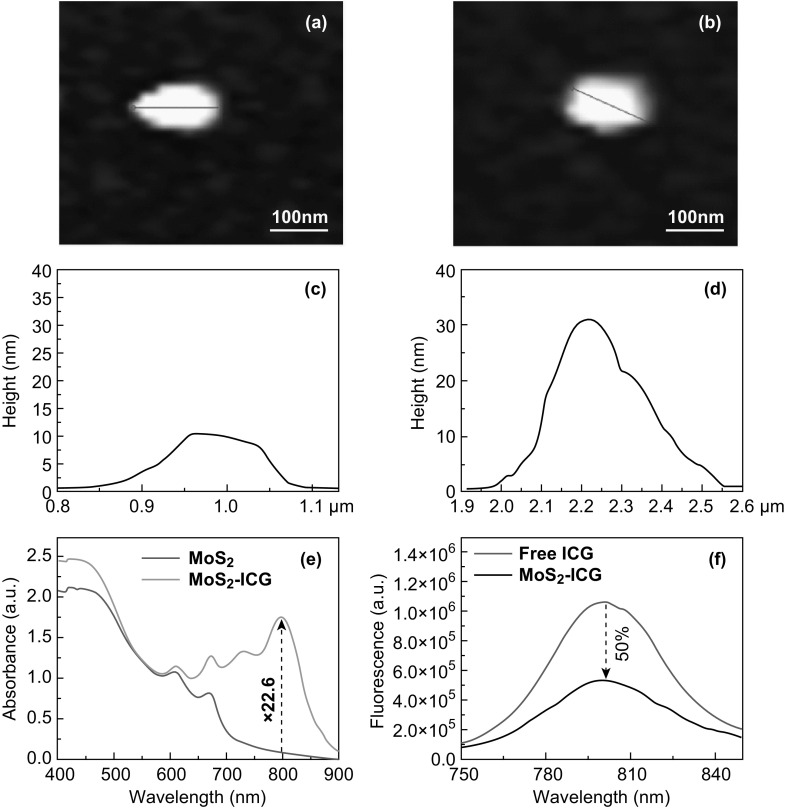
**a**, **b** AFM images and **c, d** thickness profiles of MoS_2_ (**a, c**) and MoS_2_–ICG (**b, d**). **e** Absorbance spectra of MoS_2_ and MoS_2_–ICG at the same concentration of MoS_2_. **f** Fluorescence spectra of free ICG and MoS_2_–ICG at the same concentration of ICG

Albumin-capped MoS_2_ nanosheets with single-layer nanostructure were prepared by protein-assisted exfoliation [22]. The obtained MoS_2_ nanosheets were conjugated with ICG through EDC crosslinking reaction, and the products were further purified by 24-h dialysis. Compared to ICG and MoS_2_ alone, a more intense peak at ~ 1650 cm^−1^ was observed from the FTIR spectrum of MoS_2_–ICG (Fig. S2), indicating the presence of a –NH–CO–bond between MoS_2_ and ICG and successful covalent conjugation of ICG to BSA via an esterification reaction [29]. The morphology and nanostructures of MoS_2_–ICG hybrid were investigated by AFM and TEM imaging. As shown in Figs. 1a–d and S3, the prepared MoS_2_–ICG showed a sheet-like morphology, and the edge thickness of both MoS_2_–ICG and MoS_2_ was 0.65 nm, revealing a single-layer structure [22, 32]. The middle thickness of MoS_2_–ICG increased from 11.2 to 32.5 nm compared to MoS_2_ nanosheets, indicating the success of ICG conjugation. The size distribution of MoS_2_–ICG was 50–250 nm, with an average diameter of 122 nm (Fig. S4). The polydispersity index and zeta potential of MoS_2_–ICG were 0.208 and − 24.6 mV, respectively (Fig. S5). The average diameter of MoS_2_–ICG did not change over 7 days in water, PBS, FBS, and cell media (Fig. S6), or at pH 6.8 condition (Fig. S7), demonstrating the good stability of the hybrid.

The ICG loading efficacy of MoS_2_–ICG was calculated to be 23.5%, which is higher than that of human serum albumin encapsulated nanoparticles (11%) [28], demonstrating that MoS_2_ with unique layer nanostructure and high large specific surface area favored the absorption of small optical dyes. The optical properties of MoS_2_ nanosheets were significantly altered after the loading of ICG. As shown in Fig. 1e, the optical absorbance of MoS_2_–ICG at the peak wavelength (800 nm) was significantly higher than that of MoS_2_ (Fig. 1e) because of the conjugation of ICG. (ICG loading efficiency was calculated to be 23.5%.) The absorbance intensity of MoS_2_–ICG was 22.6-fold (vertical dashed line in Fig. 1e) higher than that of MoS_2_ nanosheets under the same concentration. In addition, the NIR absorption spectrum of MoS_2_–ICG was expanded compared to that of ICG, with a redshift of the absorbance peak by 20 nm (from 780 to 800 nm) (Fig. S8). The broadened NIR spectrum and redshift of the absorbance peak occurred presumably because of the covalent conjugation of ICG onto the MoS_2_ surface, resulting in local aggregation of ICG molecules into oligomers [33–35]. After conjugating ICG to MoS_2_ nanosheets, the fluorescence of MoS_2_–ICG decreased by nearly 50% compared to free ICG at the same concentration of ICG (Fig. 1f), likely due to the fluorescence quenching effect induced by ICG aggregation and FRET (fluorescence/Förster resonance energy transfer) photoacoustic effect as reported previously [29, 36–40]. The decreased fluorescence intensity leads to greater photothermal conversion. Therefore, the photothermal/photoacoustic conversion efficiency of MoS_2_–ICG was enhanced compared to that of free ICG.

### Photoacoustic Properties of MoS_2_–ICG Hybrid

**Fig. 2:**
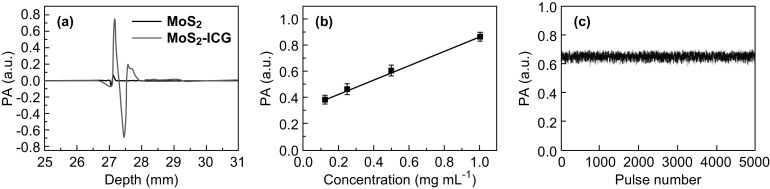
**a** Photoacoustic (PA) signal comparison of MoS_2_ and MoS_2_–ICG at the same concentration of MoS_2_. Photoacoustic signals of **b** MoS_2_–ICG at different concentrations and **c** MoS_2_–ICG illuminated with 5000 laser pulses

The photoacoustic properties of MoS_2_–ICG were investigated with our custom-built AR-PAM system [22]. As shown in Fig. 2a, the photoacoustic signal of MoS_2_–ICG was nearly 16-fold higher than that of MoS_2_ under 800-nm laser excitation at the same concentration of MoS_2_, suggesting that the conjugation of ICG significantly enhanced the photoacoustic sensitivity of MoS_2_ nanosheets. Figure 2b shows that the photoacoustic signal of MoS_2_–ICG was linearly dependent on its concentration (*R*^2^= 0.99) when the concentration range was between 0.125 and 1 mg mL^−1^. No obvious photoacoustic signal attenuation was detected after MoS_2_–ICG was irradiated with 5000 laser pulses with 5 mJ cm^−2^ laser fluence (Fig. 2c), indicating the excellent photostability of the hybrid.

**Fig. 3:**
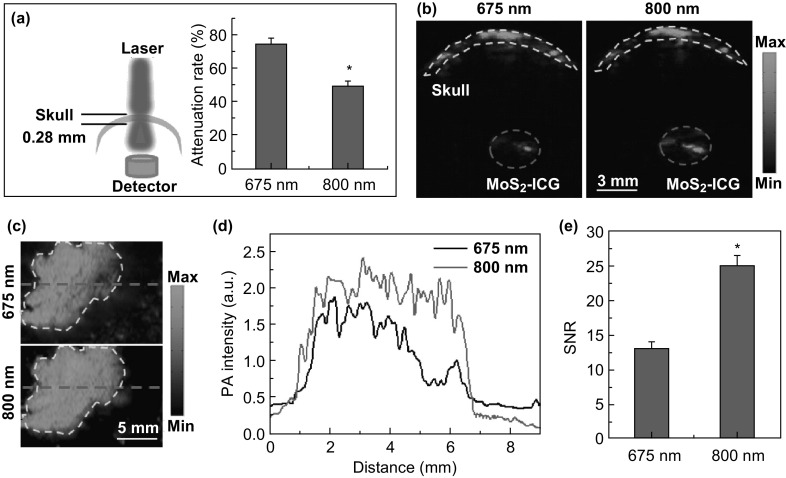
**a** Attenuation rate of 675- and 800-nm pulsed laser after penetrating the mouse skull. **b** Photoacoustic cross-sectional B-scan images of MoS_2_–ICG covered with mouse skull under 675- and 800-nm laser excitation. Yellow dashed line delineates the outline of the skull. Red circle indicates the MoS_2_–ICG sample. **c** Photoacoustic MAP images of mouse back post-subcutaneous injection of MoS_2_–ICG under 675- and 800-nm laser excitation. The enclosed area by the yellow dashed line indicates the injected region. **d** Photoacoustic signal intensity plot corresponding to the two red dashed lines in **c**. **e** SNR of photoacoustic images under 675- and 800-nm laser excitation in **c**

The laser energy attenuation for both 675- and 800-nm pulsed laser wavelengths was detected after penetrating through the mouse skull as shown in Fig. 3a. Compared to 675 nm, the 800-nm wavelength enabled greater laser energy penetration, mainly because of decreased optical scattering of the skull at the longer wavelength. Figure 3b shows the photoacoustic cross-sectional B-scan images of MoS_2_–ICG covered with the mouse skull under both 675- and 800-nm pulsed laser excitation at the same illumination energy. A higher photoacoustic signal was observed at 800-nm compared to that at 675 nm, mainly due to the more penetrated laser energy and higher optical absorbance at that wavelength. Further, the MoS_2_–ICG mixed with Matrigel matrix was subcutaneously injected into the mouse back and excited by 675- and 800-nm pulsed laser to obtain the photoacoustic maximum amplitude projection (MAP) images as shown in Fig. 3c. To quantify the in vivo photoacoustic performance with the two excitation wavelengths, the same dashed line was drawn on the two MAP images in Fig. 3c, and the photoacoustic signal intensity along the dashed line was plotted (Fig. 3d). The peak intensity of 800-nm wavelength was approximately 1.35-fold higher than that of 675 nm, which is consistent with the absorbance difference at the two wavelengths. Figure 3e further shows the SNR comparison of the photoacoustic MAP images at the two wavelengths. The 1.55-fold higher SNR for 800-nm excitation attributed to not only the higher photoacoustic signal intensity of MoS_2_–ICG at this wavelength (Fig. 3d), but also the lower background noise (Fig. 3c, d), as less tissue absorption and scattering occurred at the 800-nm wavelength. Thus, it is indicated that 800-nm pulsed laser is more suitable for in vivo MoS_2_–ICG enhanced photoacoustic imaging of brain glioma.

### Cellular Uptake and in Vitro Biocompatibility of MoS_2_–ICG Hybrid

**Fig. 4:**
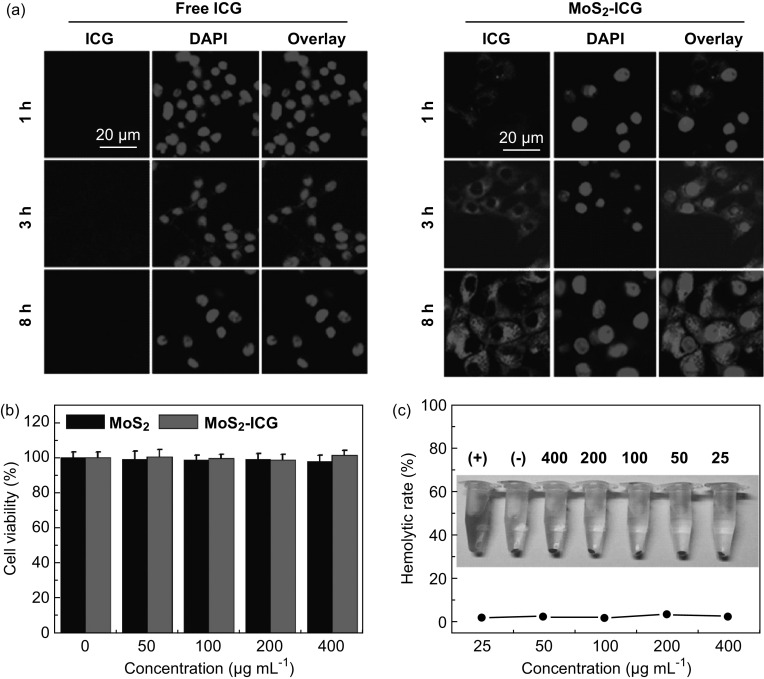
**a** Confocal fluorescence images of U87 glioma cells incubated with free ICG and MoS_2_–ICG for 1, 3, and 8 h. Blue shows fluorescence of DAPI and red shows fluorescence of ICG. **b** Viability of U87 glioma cells incubated with different concentrations of MoS_2_–ICG for 24 h. **c** Hemolysis percentage of RBCs after treatment with different concentrations of MoS_2_–ICG for 3 h. (+) and (−) each indicates distilled water and PBS as positive and negative controls. The inset photograph shows the direct observation of hemolysis

The cellular uptake behavior of MoS_2_–ICG hybrid was investigated by confocal microscopy. ICG fluorescence signals were observed in the cytoplasm of MoS_2_–ICG-treated U87 glioma cells at different time points (1, 3, and 8 h). As shown in Fig. 4a, numerous MoS_2_–ICG nanoprobes entered the cells and centered at the cytoplasm. The enhanced cellular uptake and internalization of MoS_2_–ICG occurred presumably through the albumin receptor-mediated endocytosis pathway in U87 glioma cells, which improved cell uptake efficiency and enhanced tumor cell targeting [22]. As shown in Fig. 4b, the cytotoxicity of MoS_2_–ICG and MoS_2_ on cells was determined by the standard CCK-8 assay. No obvious cytotoxicity was observed in U87 glioma cells after treatment with different concentrations (50, 100, 200, and 400 μg mL^−1^) of MoS_2_–ICG and MoS_2_ nanosheets. Moreover, the effect of MoS_2_–ICG on the hemolytic behavior of RBCs was also investigated and is shown in Fig. 4c. PBS and deionized water were used as negative and positive controls, respectively. The hemolysis percentages were lower than 2.5% at all tested MoS_2_–ICG concentrations from 25 to 400 μg mL^−1^ (Fig. 4c), indicating that MoS_2_–ICG did not induce hemolysis. These result demonstrated that MoS_2_–ICG had good biocompatibility in vitro.

### In Vivo Photoacoustic Imaging of MoS_2_–ICG in Orthotopic Glioma Model

**Fig. 5:**
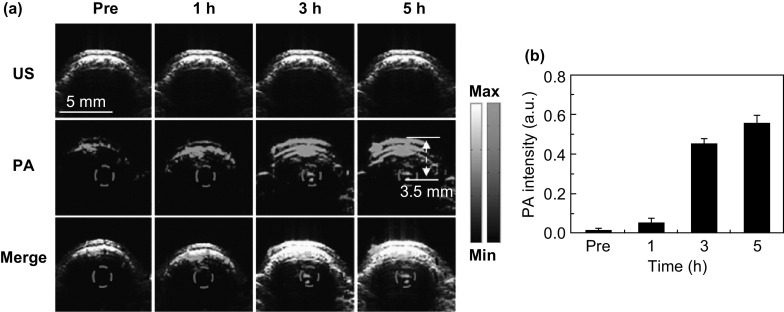
**a** B-scan ultrasound (US), photoacoustic (PA), and their merged images of the brain tumor region before and at 1, 3, and 5 h after intravenous injection of MoS_2_–ICG. The ultrasound images were used to delineate the scalp and skull of the mouse. The photoacoustic signals in the red circles show the accumulation and distribution of MoS_2_–ICG within the brain glioma. **b** Quantification results of photoacoustic signals in the tumor region at different time points before and after MoS_2_–ICG injection

The success of tumor model establishment was confirmed by the MRI results (Fig. S9). Figure 5a shows the cross-sectional (B-scan) ultrasound, photoacoustic, and their merged images of the tumor region before and after the tail-vein injection of MoS_2_–ICG. The ultrasound images were used to provide the boundary information of the tumor, as well as to confirm the site of the tumor during the experiment. The photoacoustic images further show the depth resolved distribution of MoS_2_–ICG in the tumor region at high resolution. The merged images therefore contain both structural and molecular information, which is vital for a variety of medical applications such as imaging guided photothermal therapy of tumors. As shown in Fig. 5a, photoacoustic signals of the blood vessels in the mouse scalp, skull, and brain were observed prior to MoS_2_–ICG injection (Pre in Fig. 5a). No obvious photoacoustic signals were observed at the tumor site (red circle) before and 1 h after MoS_2_–ICG injection. However, at 3 and 5 h post-injection, photoacoustic signals were clearly visualized at the tumor site because of the accumulation of MoS_2_–ICG. The multiple (three) layers at 3 and 5 h in Fig. 5a correspond to the contrast agent-enhanced scalp, skull, and brain cortex. Quantitative analysis of the photoacoustic signal enhancement in the tumor region at different time points post-injection was also performed. As can be seen from Fig. 5b, the photoacoustic signal gradually increased from 1 to 5 h after the injection of MoS_2_–ICG, indicating more and more accumulation of the hybrid in the tumor region. The specific accumulation of MoS_2_–ICG in tumor was presumably due to the enhanced permeability and retention (EPR) effect of the tumor and the albumin receptor-mediated tumor targeted effect of the MoS_2_–ICG hybrid. The hybrid has an average diameter of 122 nm, which is in accordance with the report that nanoparticles with average size in range of 10–200 nm possess better EPR effect in solid tumor [41]. In addition, the BSA coating on the surface of MoS_2_–ICG endows the hybrid with great biocompatibility, resulting in prolonged blood circulation time and more tumor accumulation. The maximum imaging depth of the tumor site was measured as 3.5 mm beneath the scalp, which can be seen from both the photoacoustic and the MRI cross-sectional images (Figs. 5a and S9b). Compared with our previous study that used MoS_2_ as contrast agent, the imaging depth in this study is enhanced by twofold. Furthermore, to the best of our knowledge, the photoacoustic imaging depth reported in this study is one of the deepest for all the photoacoustic glioma imaging research reported so far by using the nanoprobe in the NIR I spectral region (see Table S1). Table S1 illustrates the comparison of the imaging depth and applied laser wavelength for the reported glioma photoacoustic imaging studies using various types of nanoparticles in the NIR I spectral region [20–22, 42–45]. It can be seen that by using the highly sensitive MoS_2_–ICG hybrid reported in this study and an excitation wavelength at 800 nm, the imaging depth as large as 3.5 mm has been reached.

### In Vivo Biocompatibility of MoS_2_–ICG

**Fig. 6:**
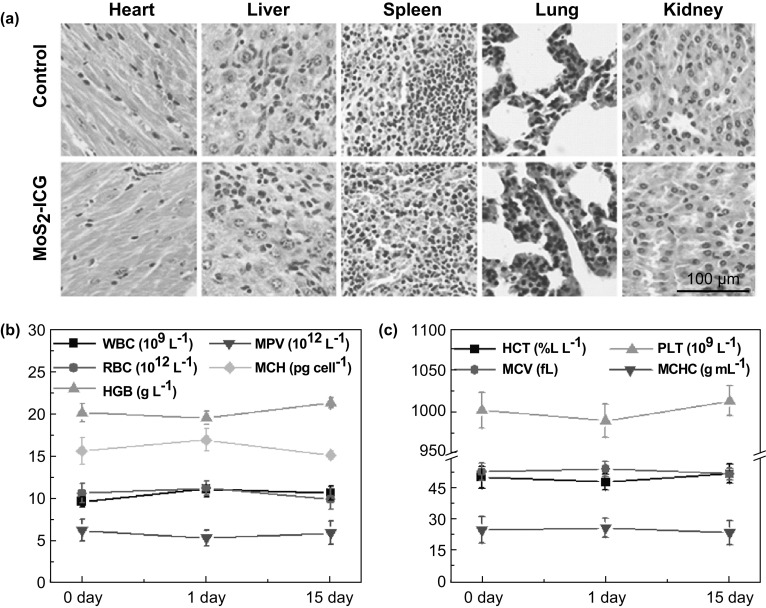
**a** Representative H&E-stained images of the major organs including the heart, liver, spleen, lung, and kidney collected from control group mice and mice treated with MoS_2_–ICG. **b**, **c** Blood analysis of MoS_2_–ICG-treated group mice. *WBC* number of white blood cells; *RBC* number of red blood cells; *MPV* mean platelet volume; *MCH* mean corpuscular hemoglobin; *HGB* concentration of hemoglobin; *HCT* hematocrit; *MCV* mean corpuscular volume; *MCHC* mean corpuscular hemoglobin concentration; *PLT* platelets

Histology analysis and a blood assay were used to evaluate the in vivo biocompatibility of MoS_2_–ICG. H&E staining images of the major organs, including the heart, liver, spleen, lung, and kidney, revealed no obvious damage or inflammation in the MoS_2_–ICG-treated group and control group (Fig. 6a). Moreover, no significant difference in blood panel parameters between the MoS_2_–ICG-treated group and control group was found (Fig. 6b, c), indicating that MoS_2_–ICG did not affect normal blood function with excellent histocompatibility. The great in vivo biocompatibility was likely due to the endogenic protein BSA coating on the MoS_2_ nanosheets.

## Conclusions

In summary, a MoS_2_–ICG hybrid was successfully prepared and applied for in vivo photoacoustic imaging of deep-sitting orthotopic brain glioma. Covalent conjugation of ICG and MoS_2_ is facile by mixing ICG-Sulfo-NHS and monolayer MoS_2_ nanosheets. The high imaging sensitivity of MoS_2_–ICG was validated, and potential causes were investigated and found to be: (1) strong optical absorbance across a broad NIR spectrum, enabling high photoacoustic signal generation; (2) redshifting of the MoS_2_–ICG absorption peak, enabling deeper penetration and lower background for in vivo imaging applications and (3) reduced ICG fluorescence due to the aggregation induced fluorescence quenching and FRET photoacoustic effect, enabling more energy to be converted to photoacoustic signal emission. Cellular uptake experiments showed that MoS_2_–ICG was internalized into the cytoplasm of U87 glioma cells with high efficiency. Both in vitro and in vivo studies showed that MoS_2_–ICG has excellent biocompatibility. In vivo photoacoustic imaging of orthotopic brain glioma demonstrated that the tumor mass sitting 3.5 mm below the scalp can be clearly identified through the enhancement by MoS_2_–ICG, which is nearly twofold deeper than that in our previous report using MoS_2_ nanosheets and to the best of our knowledge, is one of the deepest among all the glioma photoacoustic imaging studies reported so far by using the nanoprobe in the NIR I spectral region. Notably, the depth of photoacoustic molecular imaging depends on both the sensitivity of the imaging probes and the performance of the imaging system. While the effort for pursuing novel imaging probes with higher sensitivity should be a sustained ongoing process, photoacoustic imaging implementation with centimeter penetration depth capability such as photoacoustic computed tomography system should also be employed to translate the current study further on bigger animal models or even human beings. To conclude, the distinctive performance of MoS_2_–ICG, combined with the unique capability of photoacoustic imaging, reveals its potential for highly sensitive and accurate glioma detection in future translational medicine.

## Electronic supplementary material

Below is the link to the electronic supplementary material.

Supplementary material 1 (PDF 644 kb)
