# Supplementary material for: Highly Sensitive MoS2–Indocyanine Green Hybrid for Photoacoustic Imaging of Orthotopic Brain Glioma at Deep Site
Source: Nanomicro Lett. 2018 Apr 23;10(3):48. doi: 10.1007/s40820-018-0202-8 (PMC6199097; doi:10.1007/s40820-018-0202-8)
Supplement: Supplementary file 1 — Supplementary material 1 (PDF 644 kb) [file 40820_2018_202_MOESM1_ESM.pdf]

Supporting Information for

Highly Sensitive MoS_2-Indocyanine Green Hybrid for Photoacoustic Imaging of Orthotopic Brain Glioma at Deep Site

Chengbo Liu[1,‡], Jingqin Chen[1,2, ‡], Ying Zhu[1,‡], Xiaojing Gong[1], Rongqin Zheng[3], Ningbo Chen[1], Dong Chen[1], Huixiang Yan[1], Peng Zhang[5], Hairong Zheng[4], Zonghai Sheng[4,*], Liang Song[1,*]

[1]Research Laboratory for Biomedical Optics and Molecular Imaging, Shenzhen Key Laboratory for Molecular Imaging, Institute of Biomedical and Health Engineering, Shenzhen Institutes of Advanced Technology, Chinese Academy of Sciences, Shenzhen 518055, People's Republic of China

[2]Shenzhen College of Advanced Technology, University of Chinese Academy of Sciences, Shenzhen 518055, People's Republic of China

[3]Department of Medical Ultrasound, The Third Affiliated Hospital of Sun Yat-sen University, Guangzhou 510630, People's Republic of China

[4]Paul C. Lauterbur Research Center for Biomedical Imaging, Institute of Biomedical and Health Engineering, Shenzhen Institutes of Advanced Technology, Chinese Academy of Sciences, Shenzhen 518055, People's Republic of China

[5]Translational Medicine R&D Center, Institute of Biomedical and Health Engineering, Shenzhen Institutes of Advanced Technology, Chinese Academy of Sciences, Shenzhen 518055, People's Republic of China

[‡]These authors contributed equally to this work.

*Corresponding authors. E-mail: zh.sheng@siat.ac.cn (Zonghai Sheng); liang.song@siat.ac.cn (Liang Song)

Supplementary Figures and Table

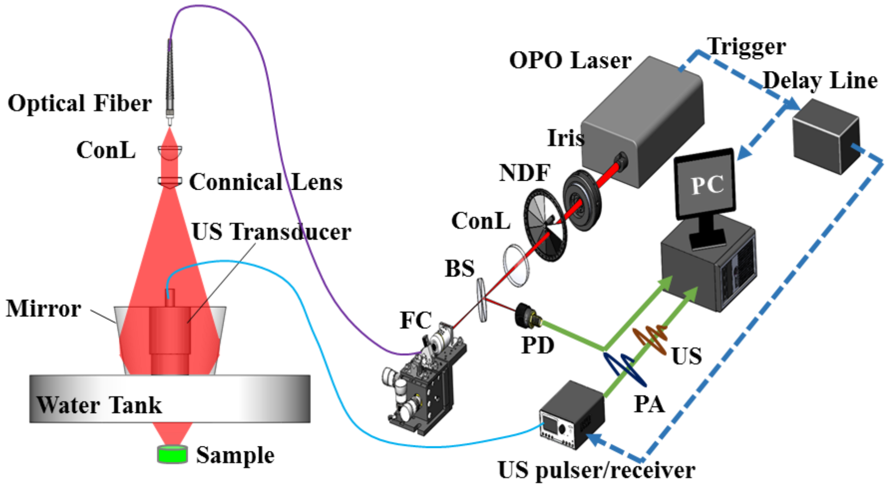

Fig. S1 Schematic illustration of the custom-built acoustic-resolution photoacoustic microscopy (AR-PAM) system. OPO: optical parametric oscillator; BS: beam splitter; PD: photodiode; FC: optical fiber coupler; PA: photoacoustic; US: ultrasound; ConL: convex lens; PC: personal computer. The OPO laser was reshaped by Iris, and then focused by a convex lens before coupling into an optical fiber via a coupler. A beam sampler and a photodiode were used to monitor the fluctuation of laser energy per pulse. The laser beam coming out of the fiber was collimated by another convex lens and focused by a conical lens to create a cone-shaped beam, which was able to pass through the ultrasound transducer and reflected by a custom-made mirror to illuminate the imaging objects. For system control, the trigger-out signal from the OPO laser was used to trigger the data acquisition board to acquire photoacoustic signals first, and a delay line was added to the trigger-out signal to trigger an ultrasonic pulser-receiver to emit an ultrasound pulse and subsequently receive the echo signals by the transducer. Both the photoacoustic and the ultrasound signals were amplified using the ultrasonic pulser-receiver, and then digitized with the DAQ in a personal computer. The motion controller manages a precision scanning stage to motorize the imaging head (dashed box) for 2D raster scanning.

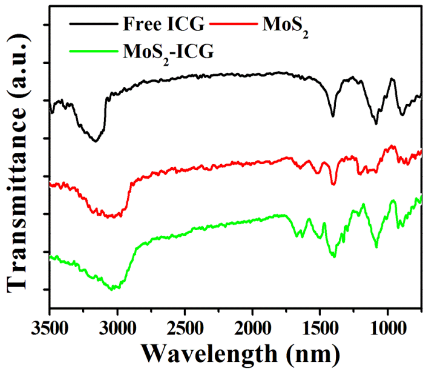

Fig. S2 FT-IR spectra of free ICG and MoS_2 and MoS_2-ICG

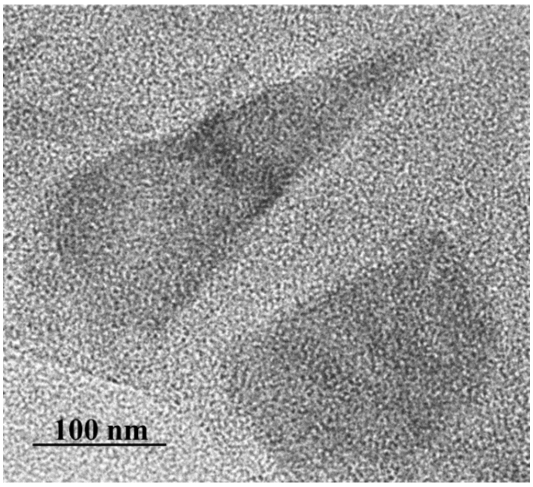

Fig. S3 TEM image of MoS_2-ICG

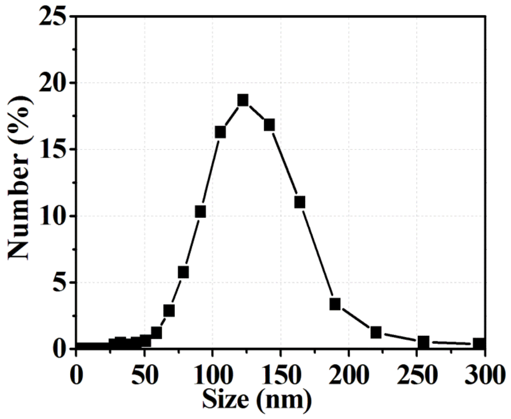

Fig. S4 Size distribution of MoS_2-ICG

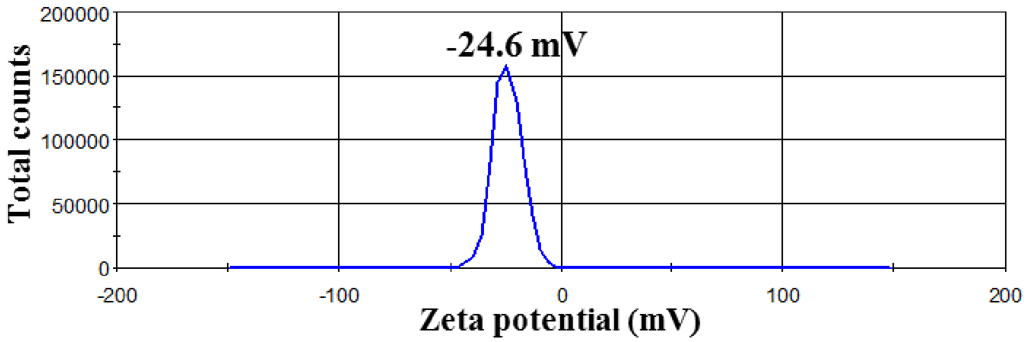

Fig. S5 Zeta potential distribution of MoS_2-ICG

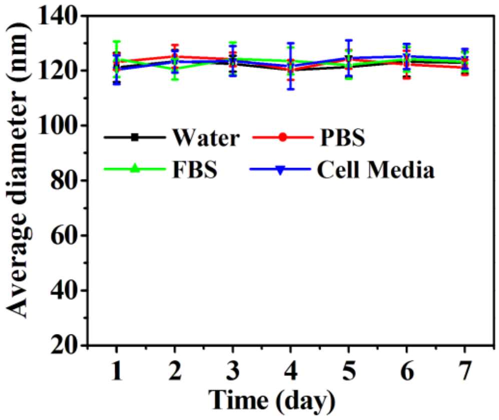

Fig. S6 Average size change of MoS_2-ICG in several media including water, PBS, FBS and cell media

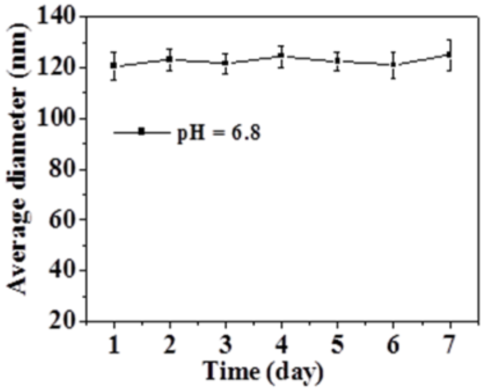

Fig. S7 Average diameter change of MoS_2-ICG in pH 6.8 condition

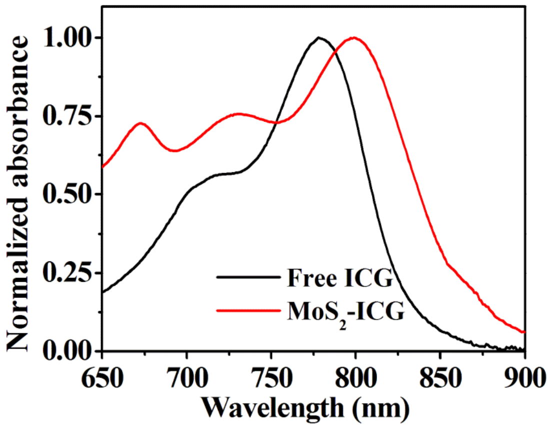

Fig. S8 Normalized absorbance spectrum of MoS_2-ICG and ICG

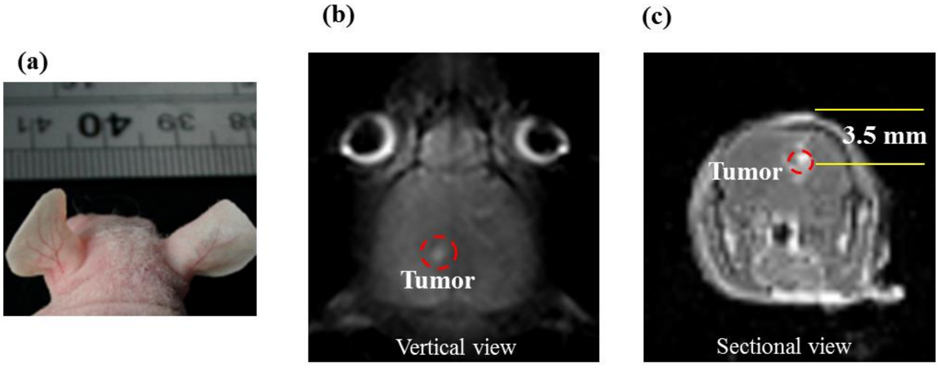

Fig. S9 a Photo of orthotopic brain glioma bearing mouse. (**b, c**) MRI images of orthotopic brain glioma, **b** is MAP image, **c** is cross sectional image. Red dashed line delineates the tumor region

Table S1 The comparison of the imaging depth and applied laser wavelength for the reported photoacoustic brain tumor imaging studies using various types of nanoparticles in the NIR I spectral region

**Types of Nanoparticles**	**Wavelength (nm)**	**Depth (mm)**	**Craniotomy**	**References**
Gold-silica based NPs	532	~3	/	[1]
Perylene-Diimide-Based NPs	700	3-4	/	[2]
Coomassie Blue-loaded NPs	590	/	Yes	[3]
HAuNS	800	/	/	[4]
IRDye800-c(KRGDf)	804	3	/	[5]
Prussian blue NPs	800	/	/	[6]
MoS_2 nanosheets	675	1.8	/	[7]
MoS_2-ICG	800	3.5	/	This work

"/": no data or ambiguity in literature
